# Recombinant XBB.1.5 boosters induce robust neutralization against KP.2- and KP.3-included JN.1 sublineages

**DOI:** 10.1038/s41392-025-02139-5

**Published:** 2025-01-27

**Authors:** Jingyun Yang, Xuemei He, Huashan Shi, Cai He, Hong Lei, Heng He, Li Yang, Wei Wang, Guobo Shen, Jinliang Yang, Zhiwei Zhao, Xiangrong Song, Zhenling Wang, Guangwen Lu, Jiong Li, Yuquan Wei

**Affiliations:** Laboratory of Aging Research and Cancer Drug Target, State Key Laboratory of Biotherapy and Cancer Center, National Clinical Research Center for Geriatrics, West China Hospital, Sichuan University, No. 17, Block 3, Southern Renmin Road, Chengdu, Sichuan 610041 People’s Republic of China

**Keywords:** Vaccines, Adaptive immunity

## Abstract

The newly emerged variants of severe acute respiratory syndrome coronavirus-2 (SARS-CoV-2) demonstrate resistance to present therapeutic antibodies as well as the capability to evade vaccination-elicited antibodies. JN.1 sublineages were demonstrated as one of the most immune-evasive variants, showing higher neutralization resistance compared to XBB.1.5. In this study, serum samples were collected from adult participants including those who had gone through the BA.5/BF.7, EG.5/HK.3 and XBB/JN.1 infection waves, characterized by different infection and vaccination histories. We evaluated the neutralization in these serum samples against pseudoviruses of Omicron lineages. We further investigated humoral immune response of recombinant XBB vaccines against Omicron variants and estimated the neutralization resistance of JN.1 sublineages, including KP.2 and KP.3. Our results showed that sera from previous circulating Omicron subvariant breakthrough infections exhibited low neutralization against pseudoviruses of Omicron lineages. The GMTs of 50% neutralization against all tested pseudoviruses were significantly elevated in sera from individuals who received WSK-V102C or WSK-V102D boosters. Importantly, the GMTs of 50% neutralization in serum samples from individuals 4 months after a WSK-V102D booster against XBB.1.5, JN.1, JN.1.13, KP.2 and KP.3 pseudoviruses were 3479, 1684, 1397, 1247 and 1298, with 9.86-, 9.79-, 8.73-, 8.66- and 8.16-fold increase compared to those without booster, respectively, indicating that boosting with XBB.1.5 subunit vaccines still induced strong antibody responses against JN.1 sublineages. However, JN.1 sublineages, including KP.2 and KP.3, revealed more than 2-fold decreases in neutralizing antibody titers compared to XBB.1.5, suggesting significantly enhanced neutralization evasion and the necessity of boosters based on JN.1, KP.2 or KP.3.

## Introduction

Since the onset of the Coronavirus Disease 2019 (COVID-19) pandemic in late 2019, over 777 million confirmed cases have been reported according to the World Health Organization (WHO), including over 7.07 million deaths as of December 2024. During the past four years, the severe acute respiratory syndrome coronavirus-2 (SARS-CoV-2) has undergone evolution, resulting in the emergence of numerous variants, including variants of concern (VOCs) Alpha (B.1.1.7), Beta (B.1.351), Gamma (P.1), Delta (B.1.617.2) and Omicron (B.1.1.529).^1,2^ The Omicron variant was first identified in South Africa near the end of 2021, and it recorded the shortest interval period of designating a variant from variants under monitoring (VUMs) to VOCs due to the largest number of mutation sites of all VOCs, accompanying with high transmissibility and potential for immune evasion.^2,3^ In the recent two years, Omicron has diversified into several different sublineages, including BA.2.75, BA.4/5, BF.7, XBB, BA.2.86 and JN.1.^4,5^ And Omicron sublineages are still the overwhelmingly dominant variants worldwide until now.

**Fig. 1:**
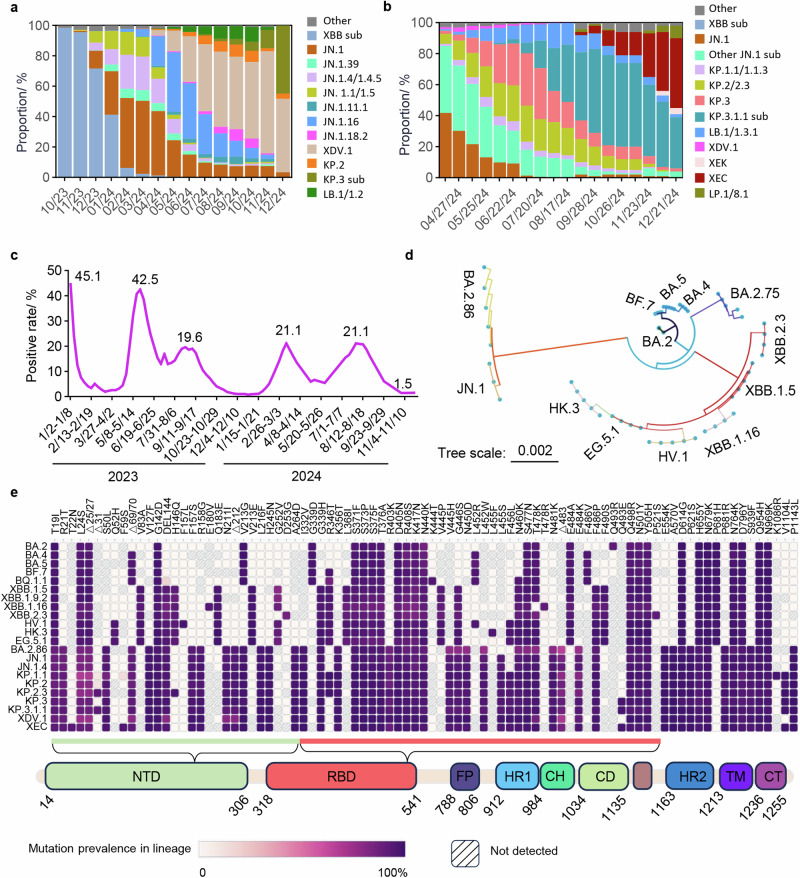
Characteristics of the JN.1 and its descendants. **a** The relative frequencies of SARS-CoV-2 lineages over time in China. Data were obtained from the GISAID database (gisaid.org/phylodynamics/china-cn/). **b** The relative frequencies of SARS-CoV-2 lineages over time in the USA. Data were obtained from CDC’s Nowcast estimates (covid.cdc.gov/covid-data-tracker/#variant-proportions). **c** Trend of the rate of SARS-CoV-2 positive in influenza-like cases in sentinel hospitals nationwide in China. Data were obtained from the Chinese Center for Disease Control and Prevention (chinacdc.cn/jkzt/crb/zl/szkb_11803/). **d** Phylogenetic tree of JN.1 and other Omicron sublineages. Sequences were downloaded from the NCBI and GISAID databases (Table S1). Evolutionary analyses were conducted in MEGA11 and the evolutionary history of a total of 59 spike nucleotide sequences was inferred using the Neighbor-Joining method. The optimal tree is shown. The tree is drawn to scale, with branch lengths in the same units as those of the evolutionary distances used to infer the phylogenetic tree. The evolutionary distances were computed using the Maximum Composite Likelihood method and are in the units of the number of base substitutions per site. **e** The mutation frequency heatmap of JN.1 and other related lineages. Only mutations with a frequency higher than 0.75 are shown. Mutation frequency data was retrieved from the GISAID website. NTD N-terminal domain, RBD receptor-binding domain, FP fusion peptide, HR1 heptad repeat 1, CH central helix, CD connector domain, HR2 heptad repeat 2, TM transmembrane domain, CT cytoplasmic tail

JN.1, a recently widespread variant, is a descendant lineage of BA.2.86. The earliest confirmed case of JN.1 infection could date back to August 2023 in Luxembourg.^6^ It subsequently derived a series of sublineages soon, such as JN.1.13, KP.2 (JN.1.11.1.2), KP.3 (JN.1.11.1.3), LB.1 (JN.1.9.2.1), JN.1.18, JN.1.4 and JN.1.5. JN.1 and its sublineages quickly replaced the pre-existing lineages and became predominant variants in more than 40 countries and areas around the world.^6,7^ The frequency of JN.1 and its sublineages is about 1% in globally collected 97261 sequences according to GISAID in October 2023, but this number increased to 77.25% only three months later. In the USA, JN.1 and its sublineages outcompeted EG.5 at the beginning of 2024. In China, JN.1 also spread fast in December 2023, and more than 90% of the recent infections were caused by JN.1 in February 2024, which replaced the earlier EG.5 and HK.3 wave (Fig. 1a). With the emergence of JN.1 sublineages, the dominant JN.1 was gradually replaced by its subvariants. Particularly, KP.2 and KP.3, with L455S, F456L and V1104L mutations, emerged concurrently.^4^ KP.2 was first detected on 2 January 2024 in India and the earliest documented sample of KP.3 was on 11 February 2024.^8^ Both of them rapidly spread in multiple regions. Further, KP.2 and KP.3 were categorized as VUMs on 3 May 2024 by WHO for their rapid diffusion.^9^ Recent studies have revealed that the relative effective reproduction number (Re) for KP.2 is 1.22, 1.32 and 1.26 times greater than that of JN.1 in the USA, UK and Canada, respectively.^10^ The Re of KP.3 is comparable to or higher than that of KP.2.^11^ Thereafter, other JN.1 subvariants, such as LB.1, XDV.1, KP.2.3 (JN.1.11.1.2.3) and KP.3.1.1 (JN.1.11.1.3.1.1) have appeared and spread quickly as of June, 2024, all of which independently developed a Serine deletion at position 31 in Spike.^12^ Notably, the Re value of KP.2.3 was even higher than that of KP.2 and KP.3.^11^ According to the latest data from the GISAID, KP.2, KP.3 and their sublineages were responsible for over one in four infections in the USA, Italy, Malaysia, India, Japan and Netherlands during the last two months.^13,14^ In the USA, the estimated variant frequencies of JN.1, KP.2 and KP.3 were 30.3%, 10.8% and 3.8%, respectively, at the last week of April, 2024, but this trend was reversed soon.^15^ By September 2024, KP.2 and KP.3 sublineages accounted for over 70% of sequenced samples.^15^ Subsequently, a new subvariant of JN.1, named XEC, began spreading rapidly across the USA (Fig. 1b), rising from 5% in September 2024 to 45% by December 2024.^15^ In China, the proportion of JN.1 sublineage XDV.1 in sequenced samples gradually increased, peaking at approximately 67% in November 2024 (Fig. 1a). Subsequently, the proportions of KP.3, KP.2 and their sublineages had been growing rapidly, surpassing that of XDV.1 by December 2024.^16^ Taken together, these data collectively suggest that JN.1 sublineages remain a major risk to human health.

Phylogenetic tree analysis results showed that BA.2.86 and its sublineage JN.1 originate from BA.2, rendering BA.2.86 and JN.1 genetically distinct from the previously circulating Omicron variants XBB.1.5, EG.5.1 and HK.3 (Fig. 1d, e). BA.2.86 carries over 50 mutations in its spike, with 25 substitutions and a deletion in the receptor binding domain (RBD) (Fig. 1e). Several substitutions, such as K417N, L452W, N460K, S477N and E484K, have been recognized as critical for antibody recognition.^17–20^ Studies revealed that BA.2.86 exhibited significant antigenic changes, notably increased receptor binding, as well as greater fusogenicity and infectivity in lung cells relative to previous variants.^21,22^ Unexpectedly, it failed to become predominant. By contrast, JN.1, with just one extra mutation L455S in its RBD relative to BA.2.86 (Fig. 1e), has quickly become the overwhelmingly dominant variant. In comparison to its direct ancestor, the BA.2.86 variant, JN.1 demonstrates significantly enhanced immune evasion and strong resistance to antibodies targeting RBD class 1, 2 and 3, along with resistance to serum neutralizing antibodies from vaccinated individuals or subjects reinfected with XBB after BA.5/BF.7, which might be largely owing to the L455S mutation.^5,22^ In addition to L455S, KP.2 has acquired three additional mutations (R346T, F456L and V1104L) while KP.3 has gained three other mutations (F456L, Q493E and V1104L), all of which are associated with immune evasion.^23,24^ R346T mutation also appeared in previously circulating variants BQ.1.1 and XBB.1.5, while R346T and F456L emerged together in recent predominant variants EG.5.1 and HK.3. The F456L mutation represents a significant genetic alteration and is related to immune evasion triggered by previous infections or vaccines.^25^ It is frequently accompanied with the L455F mutation and is known as ‘Flip’.^26^ This combination greatly enhances the affinity of spike to ACE2, which enables the virus to better accommodate further mutations for immune evasion.^8^ However, for KP.2 and KP.3, these titers were significantly lower (about 1.6-fold to 2.2-fold) than that against JN.1 in monovalent XBB.1.5 vaccine sera and convalescent sera after XBB.1.5, EG.5, HK.3 and JN.1 infections.^10,12^ Additionally, the 50% neutralization titers for KP.2.3 were considerably reduced compared to those for KP.2, by approximately 1.4-fold to 1.7-fold.^11^ With an additional serine deletion at position 31 in the spike protein (S:S31del) compared to KP.3, KP.3.1.1 demonstrated a 1.2-fold increase in Re and increased pseudovirus infectivity. The 50% neutralization titers for KP.3.1.1 were 1.4-1.6-fold lower than those for KP.3 in convalescent serum samples.^11,12^ Furthermore, KP.3.1.1 exhibited 1.3-fold lower neutralization titers against XBB.1.5 vaccine serum samples compared to KP.3.^12^ These findings raise concerns that immunity induced by prior Omicron infections or COVID-19 vaccinations may not provide sufficient protection against KP.2, KP.3 and their sublineage infections, given their increased fitness and immune evasion capabilities.

During the period from May 2020 to December 2022, a small number of Chinese were diagnosed with COVID-19 contributing to the “dynamic zero-COVID” policy by the government.^27^ With the prevalence of Omicron BA.5 which has high transmission and reduced pathogenicity, the Chinese government modified its response strategies. Since then, Omicron infections have spread rapidly across major cities in China. There were five major waves of COVID-19 outbreaks have been recorded in China since early December 2022 according to the data from the Chinese Center for Disease Control and Prevention (Fig. 1c). Based on the rapid increase in the proportion of KP.3 and its sublineages in sequenced samples by the end of December and the trend of infection peaks shown in Fig. 1c, it is predicted that the sixth infection wave driven by KP.3 and its sublineages might arrive soon, making it crucial to evaluate whether population immunity remains protective. Therefore, systematic evaluation of the neutralization against the newly emerged Omicron subvariants in the sera of convalescents or vaccinated individuals is necessary.

In this study, we recruited 339 participants with diverse vaccination and infection histories from Chengdu, China. Since more than 80% of the population contracted BA.5/BF.7 infections between December 2022 and January 2023,^28,29^ and about one-fifth of the population was infected during the XBB wave between May to July 2023.^29^ We characterized the neutralization against primary Omicron variants in the serum samples collected in different periods and sera from vaccinated individuals who received a dose of Recombinant COVID-19 Trivalent (XBB.1.5 + BA.5+Delta) Protein Vaccine (Sf9 Cell) (WSK-V102C) in February 2023, and sera from volunteers that received Recombinant COVID-19 (XBB) Trimer Protein Vaccine (Sf9 Cell) (WSK-V102D) in October 2023. We also compared the neutralization against Omicron subvariants between the serum samples from individuals with or without booster. The data showed that sera from individuals with XBB boosters (WSK-V102C or WSK-V102D) still reserved a high level of neutralization against not only previous predominant Omicron subvariants but also current JN.1 subvariants, which suggests XBB vaccines still demonstrate promising efficacy in protecting JN.1 sublineages. However, JN.1 sublineages exerted enhanced immune evasion against sera from individuals with different vaccination and infection histories, emphasizing the necessity for ongoing vaccine development.

## Results

### Neutralization of Omicron subvariants by sera from individuals with different immune backgrounds and infection histories

**Fig. 2:**
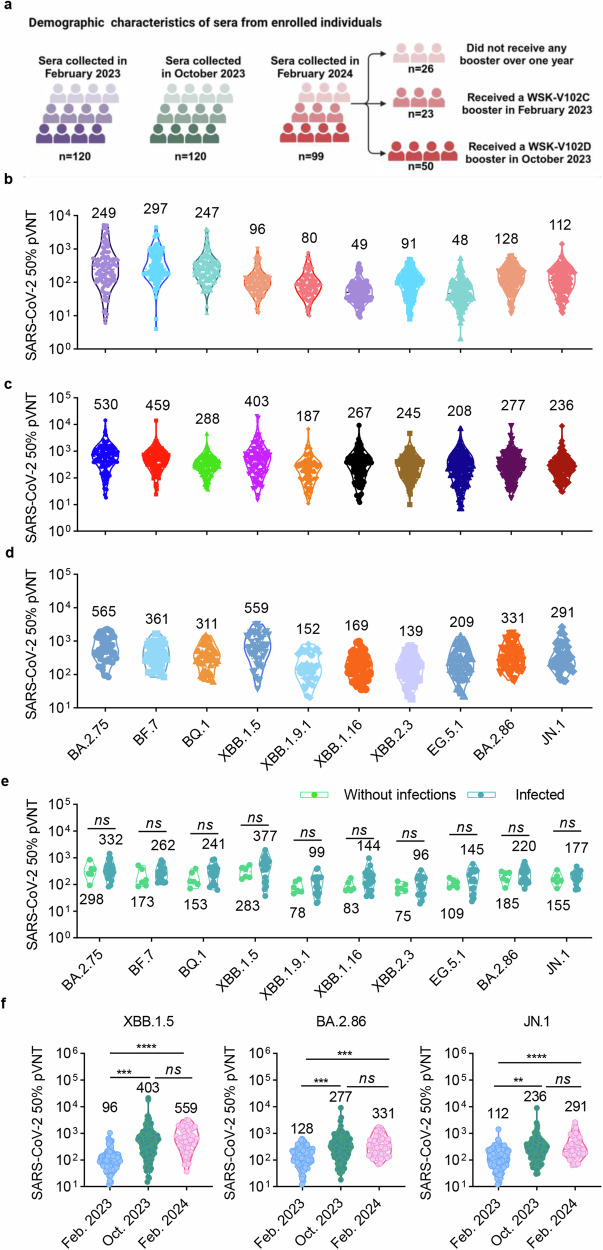
Neutralization of distinct Omicron sublineages by sera collected in three separate periods. **a** Demographic characteristics of sera from enrolled individuals. **b** Neutralizing antibody titers against BA.2.75, BF.7, BQ.1, XBB.1.5, XBB.1.9.1, XBB.1.16, XBB.2.3, EG.5.1, BA.2.86 and JN.1 pseudoviruses in sera from 120 adult participants in February 2023, 2-3 months post a BA.5/BF.7 breakthrough infection in December 2022. **c** Neutralizing antibody titers against Omicron sublineages pseudoviruses in 120 sera samples collected in October 2023. **d** Neutralization of different Omicron sublineages pseudoviruses by sera collected from 49 individuals in February 2024. **e** Neutralization against Omicron sublineage pseudoviruses by sera from participants with or without infection history. 6 of those 26 participants who did not receive a vaccine booster more than one year reported no infection history, and another 20 claimed infections, including 17 infected with BA.5/BF.7 and 3 infected during XBB infection wave. **f** Comparison of neutralizing antibody titers against XBB.1.5, BA.2.86 and JN.1 pseudoviruses in sera samples collected in three separate periods. Data are presented as geometric mean values ± SD in (**b**–**f**). Unpaired Student’s *t*-tests were performed. Statistical significance is indicated by *p < 0.05, **p < 0.01, ***p < 0.001, ****p < 0.0001, ns not significant

To assess the levels of neutralizing antibodies against Omicron subvariants in sera of the population at different time points, we collected sera from a total of 339 participants across three periods: February 2023, October 2023, and February 2024. The serum collection details are shown in Fig. 2a. We first evaluated the neutralizing antibody titers against BA.2.75, BF.7, BQ.1, XBB.1.5, XBB.1.9.1, XBB.1.16, XBB.2.3, EG.5.1, BA.2.86 and JN.1 pseudoviruses in sera from 120 adult participants collected in February 2023, 2-3 months post a BA.5/BF.7 breakthrough infection wave. The results showed that the geometric mean titers (GMTs) of 50% neutralization in sera against BA.2.75, BF.7 and BQ.1 were 249, 297 and 247, respectively (Fig. 2b). As the previous predominant variant, several studies have reported that XBB.1.5 possessed extraordinary evasion properties. Consistent with this, we discovered that the GMTs of 50% neutralization against XBB.1.5 in sera was 96 and there was a similar reduction in titers for XBB.1.9.1, XBB.1.16, XBB.2.3, EG.5.1, BA.2.86 and JN.1 pseudoviruses (Fig. 2b). In October 2023, after the XBB.1.5 and EG.5 infection waves, we collected serum samples from 120 individuals, including those infected with XBB.1.5/EG.5 or received a dose of WSK-102C vaccine in February 2023. As observed in Fig. 2b, c, the neutralizing antibody titers against Omicron subvariants were higher compared to those collected in February 2023. The GMTs of 50% neutralization against BA.2.75, BF.7, BQ.1, XBB.1.5, XBB.1.9.1, XBB.1.16, XBB.2.3, EG.5.1, BA.2.86 and JN.1 pseudoviruses were 530, 459, 288, 403, 187, 267, 245, 208, 277 and 236, respectively (Fig. 2c). As expected, the serum neutralizing titers against XBB.1.5 and EG.5.1 pseudoviruses elevated remarkably with a 4.2- and 4.3-fold increase, which is consistent with the fact that the sequences of XBB.1.5 and EG.5 are extremely similar, therefore, both XBB.1.5/EG.5 breakthrough infection and XBB.1.5 vaccine injection could induce increased neutralizing antibodies against themselves. We next determined the neutralizing antibodies in serum samples collected in February 2024, when there was a beginning of JN.1 infection wave. We found that the 50% neutralization GMTs in 49 serum samples collected in February 2024 were comparable to those collected in October 2023 (Fig. 2c, d). We then analyzed the neutralization of serum samples collected from individuals with or without infection history in February 2024, and found that the 50% neutralization titers against Omicron sublineage pseudoviruses showed no significant difference, indicating that the neutralization elicited by previous Omicron subvariant breakthrough infections is insufficient to protect against tested Omicron variants (Fig. 2e). Taken together, the 50% neutralization titers against Omicron subvariants, including XBB.1.5, BA.2.86 and JN.1, were raised after undergoing XBB infection wave (Fig. 2f). However, the titers against BA.2.86 and JN.1 pseudoviruses were still at a low level, which suggests insufficient protective effect against newly emerged JN.1 sublineages.

### WSK-V102C booster improves the neutralization against Omicron subvariant pseudoviruses

**Fig. 3:**
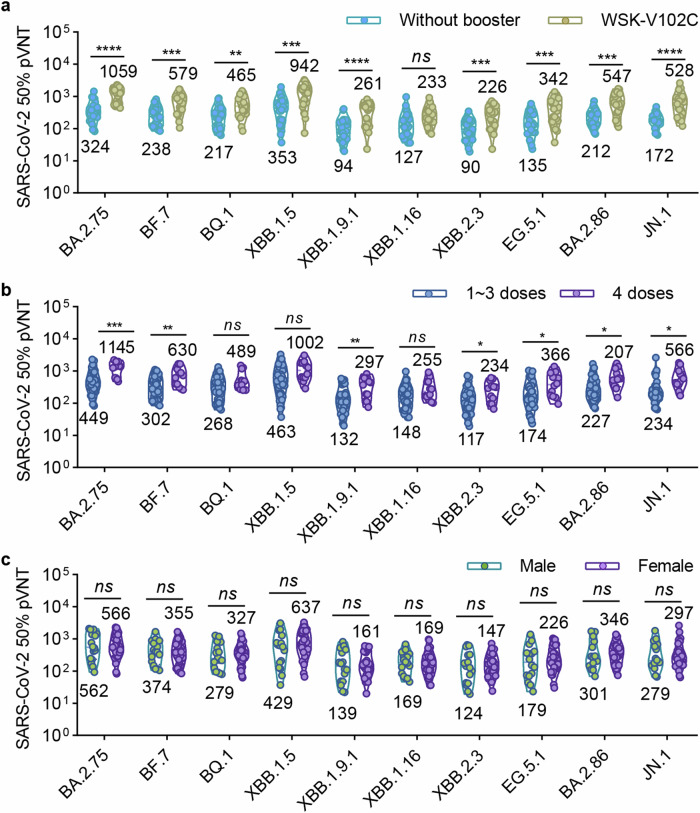
Impacts of vaccine booster and age on neutralization against Omicron sublineages. **a** Comparison of neutralizing antibody titers against BA.2.75, BF.7, BQ.1, XBB.1.5, XBB.1.9.1, XBB.1.16, XBB.2.3, EG.5.1, BA.2.86 and JN.1 pseudoviruses in sera from individuals with or without a WSK-V102C booster in the past year. Among all the 49 individuals, 26 participants did not receive a vaccine booster for more than one year, while 23 of them received a dose of WSK-V102C booster in the period around February 2023. **b** Comparison of neutralization against Omicron sublineage pseudoviruses by sera from participants who received 1 ~ 3 doses (*n* = 37) and 4 doses (*n* = 12) of COVID-19 vaccine. **c** Comparison of neutralization against different Omicron sublineage pseudoviruses by sera from male (*n* = 16) and female (*n* = 33) participants. Data are presented as geometric mean values ± SD in (**a–c**). Unpaired Student’s *t*-tests were performed. Statistical significance is indicated by *p < 0.05, **p < 0.01, ***p < 0.001, ****p < 0.0001, *ns* not significant

To evaluate the neutralizing antibody responses elicited by WSK-V102C booster over 1 year against currently circulating and newly emerged Omicron subvariants, serum samples from 26 participants who had not received any booster and 23 individuals who received a dose of WSK-V102C booster in February 2023 were collected. Table 1 provides a detailed summary of the demographics and vaccination histories of the 49 participants. The results showed that the 50% neutralization GMTs in the sera from those without any vaccine booster in the past year against BA.2.75, BF.7, BQ.1, XBB.1.5, XBB.1.9.1, XBB.1.16, XBB.2.3, EG.5.1, BA.2.86 and JN.1 pseudoviruses were 324, 238, 217, 353, 94, 127, 90, 135, 212 and 172, respectively. In contrast, sera from the individuals with one dose of WSK-V102C booster demonstrated significantly enhanced neutralizing titers against all tested pseudoviruses. The 50% neutralization GMTs raised to 1059, 579, 465, 942, 261, 233, 226, 342, 547 and 528, indicating a 3.27-, 2.43-, 2.14-, 2.67-, 2.78-, 1.83-, 2.51-, 2.53-, 2.58- and 3.07-fold increase, respectively (Fig. 3a). We found that the neutralization against Omicron sublineage pseudoviruses in sera from individuals vaccinated with 4 doses was higher than those with 1 ~ 3 doses, indicating the importance of receiving a booster derived from current circulating variant (Fig. 3b). What’s more, consistent with the previous studies, the titers in male individuals were comparable with those in females (Fig. 3c). Our data demonstrated that the neutralization titers in individuals who received a WSK-V102C booster, even one year prior, remain at a relatively high level.

**Table 1:** Demographic characteristics of enrolled individuals without WSK-V102D booster in Chengdu, China, in February 2024

	Without WSK-V102C booster (*n* = 26)	With WSK-V102C booster (*n* = 23)	Total (*n* = 49)
**Age**			
Average age	34.42 (±11.92)	35.87 (±11.64)	35.10 (±11.69)
18-59 years *n* (%)	25 (51.02%)	23 (46.94%)	48 (97.96%)
≥ 60 years *n* (%)	1 (2.04%)	0	1 (2.04%)
**Gender**			
Male *n* (%)	8 (16.33%)	8 (16.33%)	16 (32.65%)
Female *n* (%)	18 (36.74%)	15 (30.61%)	33 (67.35%)
**Number of previous COVID-19 vaccinations**			
0-3 doses *n* (%)	25 (51.02%)	12 (24.49%)	37 (75.51%)
4-6 doses *n* (%)	1 (2.04%)	11 (22.45%)	12 (24.49%)
**Heterologous boost**			
Yes *n* (%)	0	22 (44.90%)	22 (44.90%)
No *n* (%)	26 (53.06%)	1 (2.04%)	27 (55.10%)
**Infection history**			
Yes *n* (%)	21 (42.86%)	14 (28.57%)	35 (71.43%)
No *n* (%)	5 (10.20%)	9 (18.37%)	14 (28.57%)

### WSK-V102D booster induces broad-spectrum neutralizing antibodies against JN.1-included Omicron subvariants

**Fig. 4:**
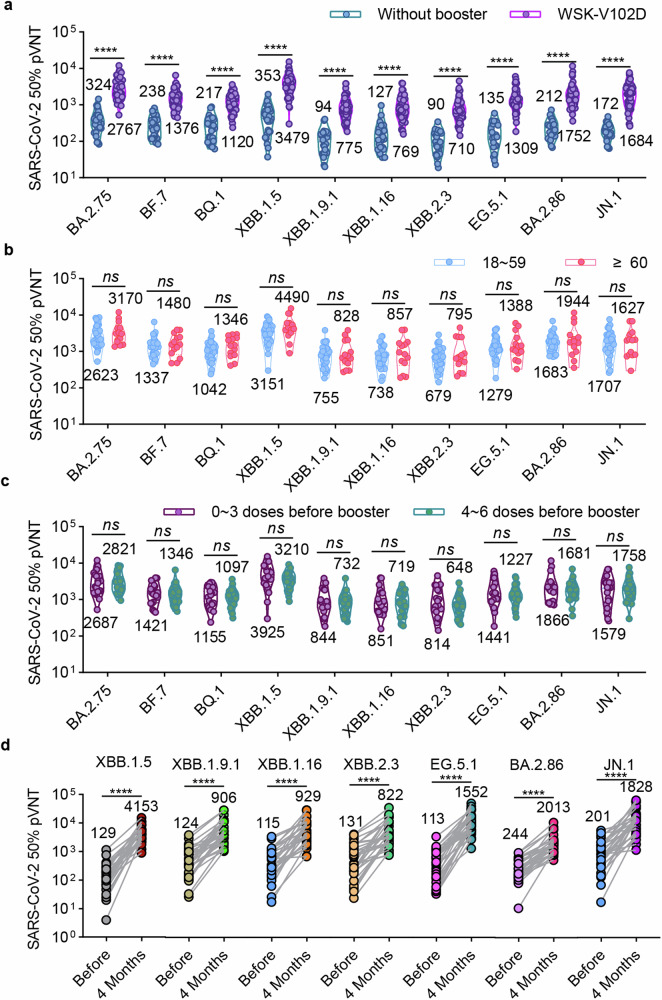
XBB vaccine booster induces high levels of broad-spectrum neutralizing antibodies against JN.1 and other omicron subvariants. **a** Comparison of neutralizing antibody titers against BA.2.75, BF.7, BQ.1, XBB.1.5, XBB.1.9.1, XBB.1.16, XBB.2.3, EG.5.1, BA.2.86 and JN.1 pseudoviruses in sera from individuals with or without XBB.1.5 vaccine booster in October 2023. 26 participants did not receive any COVID-19 vaccine booster for more than one year, and another 50 participants received a dose of WSK-V102D in October 2023. **b** Comparison of neutralization against different Omicron sublineage pseudoviruses by sera from 36 adult (18-59 group) and 14 elderly (≥60 group) participants. **c** Comparison of neutralization against Omicron sublineage pseudoviruses by sera from participants who received 0 ~ 3 doses (*n* = 20) and 4 ~ 6 doses (*n* = 30) of COVID-19 vaccines before receiving a WSK-V102D booster. **d** In parallel comparison of neutralization against distinct Omicron subvariants by 30 individual-matched serum samples collected from individuals before and 4 months after a WSK-V102D booster vaccination. Data are presented as geometric mean values ± SD in (**a–d**). Unpaired Student’s *t*-tests were performed. ****p < 0.0001, *ns* not significant

Given that the mutations in JN.1 and BA.2.86 spike are notably distinct from XBB.1.5, there is concern that previously approved XBB.1.5-related COVID-19 vaccines will not effectively protect against currently circulating JN.1 and its sublineages. Therefore, we investigated the neutralizing antibody responses induced by the Recombinant COVID-19 (XBB) Trimer Protein Vaccine (Sf9 Cell) (WSK-V102D). We recruited 50 volunteers who had received a dose of WSK-V102D booster over 4 months and collected serum samples to evaluate the neutralization against Omicron sublineages. The vaccination histories of the participants are outlined in Table 2. As shown in Fig. 4a, four months after receiving a WSK-V102D booster, increased broad-spectrum neutralizing antibodies against JN.1-included Omicron subvariants were observed. Among them, the serum 50% neutralization GMTs against XBB.1.5 were the highest, raising from 353 to 3479, followed by those against other Omicron subvariants. The 50% neutralization GMTs in sera induced by WSK-V102D booster against BA.2.75, BF.7, BQ.1, XBB.1.9.1, XBB.1.16, XBB.2.3, EG.5.1, BA.2.86 and JN.1 pseudoviruses were 2767, 1376, 1120, 775, 769, 710, 1309, 1752 and 1684, showing an 8.54-, 5.78-, 5.16-, 8.24-, 6.06-, 7.89-, 9.70-, 8.26- and 9.79-fold increase. The increase in neutralization was indistinguishable between individuals 18 to 59 years old and those over 60 years old (Fig. 4b). Moreover, the levels of neutralizing antibodies induced by WSK-V102D booster were comparable regardless of whether individuals had received 0-3 doses or 4-6 doses of COVID-19 vaccines before receiving WSK-V102D booster (Fig. 4c). To more accurately evaluate the protection of the WSK-V102D vaccine, 30 individual-matched serum samples were analyzed before and 4 months after the booster vaccination. As shown in Fig. 4d, the WSK-V102D booster significantly increased serum neutralization titers against all Omicron subvariants tested. The GMTs of 50% neutralization in sera against XBB.1.9.1, XBB.1.16, XBB.2.3 and EG.5.1 pseudoviruses were 906, 929, 822 and 1552, respectively. The highest neutralization titers were observed against XBB.1.5 (GMT = 4153), followed by BA.2.86 (GMT = 2013) and JN.1 (GMT = 1828), with a 32.19-, 8.25- and 9.09-fold increase, respectively, suggesting that boosting with WSK-V102D could be effective in protecting individuals from JN.1 wave.

**Table 2:** Demographic characteristics of individuals with WSK-V102D booster

50 adults received a dose of WSK-V102D booster in October 2023	
**Age**	
Average age	41.6 (±16.6)
18-59 years *n* (%)	36 (72.00%)
≥ 60 years *n* (%)	14 (28.00%)
**Gender**	
Male *n* (%)	15 (30.00%)
Female *n* (%)	35 (70.00%)
**Number of previous COVID-19 vaccinations before WSK-102D**	
0-3 doses *n* (%)	20 (40.00%)
4-6 doses *n* (%)	30 (60.00%)
**WSK-V102C booster**	
Yes *n* (%)	32 (64.00%)
No *n* (%)	18 (36.00%)
**Heterologous boost**	
Yes *n* (%)	50 (100.00%)
No *n* (%)	0
**Infection history**	
Yes *n* (%)	23 (46.00%)
No *n* (%)	27 (54.00%)

### JN.1 descendants exhibit enhanced neutralization evasion

**Fig. 5:**
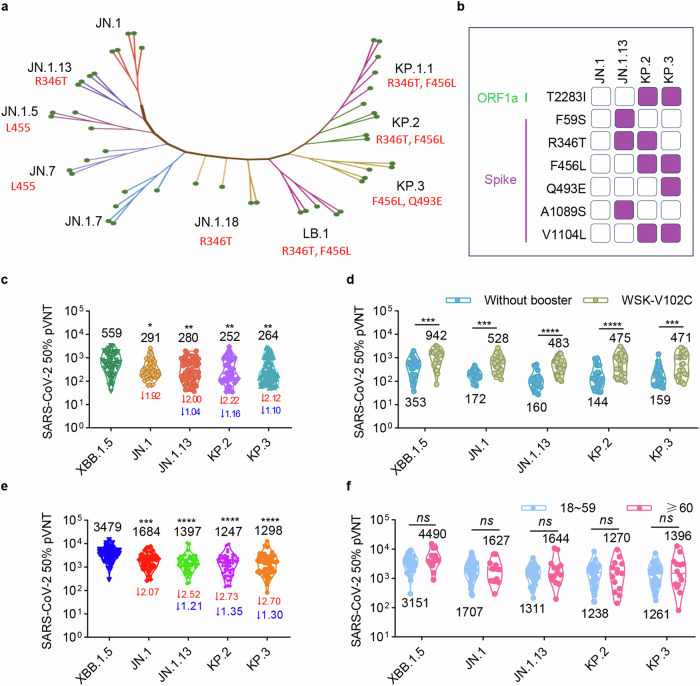
JN.1 sublineages exhibit enhanced immune evasion. **a** Phylogenetic tree of JN.1 sublineages. Sequences were downloaded from the NCBI database. Evolutionary analyses were conducted in MEGA11 using the Neighbor-Joining method. The optimal tree is shown with additional mutations in RBD compared with JN.1. **b** The comparison of mutation in spike of JN.1, JN.1.13, KP.2 and KP.3. Only mutations with a frequency higher than 0.75 are shown. Mutation frequency data was retrieved from the GISAID website. **c** Neutralization against Omicron sublineage pseudoviruses by serum samples collected from 49 individuals in February 2024. **d** Comparison of neutralizing antibody titers in sera from individuals with or without a vaccine booster in the past year. Among all the 49 individuals, 26 participants did not receive a booster for more than one year, while 23 of them received a dose of WSK-V102C in the period around February 2023. **e** Neutralization against Omicron sublineage pseudoviruses by serum samples collected from 50 participants 4 months after a dose of WSK-V102D. **f** Comparison of neutralization against JN.1 sublineage pseudoviruses by sera from 36 adult (18-59 group) and 14 elderly (≥60 group) participants. Data are presented as geometric mean values in (**c–f**). Two-way ANOVA multiple comparisons were performed. Statistical significance is indicated by *p < 0.05, **p < 0.01, ***p < 0.001, ****p < 0.0001, *ns* not significant

With rapid evolution of JN.1 sublineages, JN.1 has driven a series of descendants, including JN.1.13, JN.1.5, JN.7, JN.1.18, LB.1, KP.3, KP.2 and KP.1.1 (Fig. 5a). Most descendants of JN.1 have inherited its RBD mutations, while JN.7 has lost the L455S mutation, and other subvariants have introduced new mutations. In detail, JN.1.13 and JN.1.18 share the recurrent additional R346T that is associated with antibody resistance. LB.1, KP.2 and KP.1.1 possess the combinations of R346T and F456L substitutions while KP.3 introduces F456L and Q493E substitutions in RBD. Among these descendants of JN.1, globally spread are JN.1.13, KP.2 and KP.3. JN.1.13 harbours additional F59S, R346T and A1089S substitutions in the spike compared to JN.1. KP.2 and KP.3 share V1104L, F456L and T2283I mutations (Fig. 5b).

Previous studies have revealed that the combination of R346T, L455S and F456L might make considerable potential for immune evasion and resistance to neutralizing antibodies elicited by repeated vaccination and infection.^23,30–32^ We then examined the neutralizing antibody titers against JN.1, JN.1.13, KP.2 and KP.3 pseudoviruses in sera collected from 49 individuals in February 2024. Compared with XBB.1.5, JN.1, JN.1.13, KP.2 and KP.3 exhibited higher neutralization resistance. The 50% neutralization GMTs against JN.1, JN.1.13, KP.2 and KP.3 pseudoviruses dropped significantly by 1.92-, 2.00-, 2.22- and 2.12-fold, respectively (Fig. 5c). We further determined the neutralization in sera from individuals after a booster of WSK-V102C and WSK-V102D. Compared to the neutralizing antibody titers in sera from 26 participants who had not received any COVID-19 booster over a year, WSK-V102C vaccination improved the neutralization in serum against XBB.1.5, JN.1, JN.1.13, KP.2 and KP.3 pseudoviruses with a 2.67-, 3.07-, 3.02-, 3.30- and 2.96-fold increase, respectively (Fig. 5d). Similarly, in the sera from 50 participants who had a WSK-V102D booster 4 months before, the 50% neutralization GMTs against XBB.1.5 were 3479. The 50% neutralization GMTs against JN.1, JN.1.13, KP.2 and KP.3 pseudoviruses were increased to 1684, 1397, 1247 and 1298, but with a 2.07-, 2.52-, 2.73- and 2.70-fold reduction compared to XBB.1.5 (Fig. 5e). Moreover, the 50% neutralization GMTs against JN.1.13, KP.2 and KP.3 pseudoviruses dropped by 1.21-, 1.35- and 1.30-fold, respectively, compared with that against JN.1, indicating the impaired neutralizing activity against newly emerged KP.2 and KP.3 subvariants (Fig. 5e). Besides, the resistance of JN.1.13, KP.2 and KP.3 to neutralization by sera was independent of age (Fig. 5f). These findings suggest that administration of XBB recombinant boosters can effectively enhance serum neutralizing capacity against KP.2- and KP.3-included variants. Meanwhile, enhanced neutralization escape of JN.1.13, KP.2 and KP.3 underscores the need for boosters based on these emerging variants to address the next wave of infections.

## Discussion

Although global vaccinations and previously repeated SARS-CoV-2 infections leads to elevated antibodies in individuals, the Omicron variants persist in spreading, even with high infection rates in those vaccinated individuals.^33^ In this case, it’s uncertain whether the antibodies induced by previous Omicron infections are enough to neutralize currently circulating KP.2, KP.3 and KP.3.1.1. Therefore, this study delves into the assessment of serum neutralization capacity against the Omicron sublineages, including the emerged variant JN.1 and its subvariants JN.1.13, KP.2 and KP.3, with a pivotal focus on evaluating the antibody immune responses of sera from individuals who vaccinated wildtype/Delta vaccines over and went through BA.5/BF.7, EG.5 and HK.3 infection waves. Our results showed that breakthrough infections with previous circulating Omicron subvariants improved the 50% neutralization titers in serum. However, the reduced neutralization in sera against JN.1 and its sublineages may not suffice for protection from infection, highlighting the necessity for effective vaccines.

The evolution of viruses might commonly rely on balancing immune evasion, strong ACE2 affinity, and adequate structural flexibility. The recently emerged Omicron subvariants, such as JN.1, KP.2 and KP.3, likely share convergent mutational hotspots during their evolution, focusing on enhancing immune evasion while maintaining adequate ACE2 binding capability.^32^ In contrast to predecessor BA.2.86, most JN.1 sublineages, including JN.1.13, KP.2 and KP.3, have an additional substitution L455S in spike. L455 is situated at the binding interface between human ACE2 and the RBD, the L455S mutation decreases binding affinity between ACE2 and the RBD of JN.1 but enhances its infectivity and immune evasion ability.^4,22^ Since L455 is primarily located at the epitope of Class 1 antibodies’ receptor-binding domain, L455S mutation endows JN.1 with the ability to evade Class 1 antibodies.^5^ Moreover, S309, a class 3 antibody, has been demonstrated to effectively neutralize the majority of Omicron variants, including XBB.1.5 and EG.5.1, but was unable to neutralize BA.2.86,^34^ likely due to mutations at residues 339 and 346 of the RBD.^35^ The G339H and R346T also appeared in JN.1.13 and JN.1.18.1, suggesting resistance to Class 1 and Class 3 antibodies. KP.2 and KP.3 directly originated from JN.1. In the RBD region, both KP.2 and KP.3 introduced the F456L substitution. Additionally, KP.2 acquired an R346T mutation, while KP.3 introduced a Q493E mutation. R346T has been observed in various Omicron subvariants and is linked to an enhanced capacity to evade neutralization induced by vaccines or breakthrough infections. F456L appeared in previously dominant variants EG.5.1 and HK.3. Studies demonstrated that epistatic interactions of L455, F456 and Q493 residues could balance high ACE2 binding affinity and immune escape.^32^ The pairing of L455F and F456L forms an adjacent residue flipping, resulting in increased resistance to neutralizing antibodies and enhanced binding affinity to ACE2.^19^ Therefore, JN.1 sublineages that carry the L455S, F456L and Q493E mutations, such as KP.2 and KP.3 variants, may have evolved to surpass other Omicron subvariants by enhancing immune evasion while maintaining a beneficial binding affinity with ACE2.^32^

Given that JN.1 subvariants are notably distinct from XBB.1.5 in spike sequence, there is concern that the updated XBB.1.5 vaccines would not effectively protect against JN.1 and its sublineages. Here, we also focus on serum neutralization against the newly emerged variants, particularly JN.1, JN.1.13, KP.2 and KP.3, to assess the effectiveness of recombinant XBB vaccines. Our findings indicate that sera from individuals vaccinated with either WSK-V102C or WSK-V102D displayed significantly higher neutralizing titers against JN.1, JN.1.13, KP.2 and KP.3 compared to sera from those without XBB boosters. Previous studies revealed that Omicron XBB.1.5 monovalent mRNA vaccine showed slightly lower protection against BA.2.86 and JN.1 variants than XBB.1.5.^36^ And other updated XBB vaccines, such as SCTV01E, WSK-V102C and XBB.1.5 mRNA vaccine, still exhibited promising efficacy in producing high neutralizing titers against a spectrum of Omicron variants, including BA.2.86 and JN.1.^29,37–39^ A Phase 2/3 Trial data demonstrated that the monovalent XBB.1.5-adapted BNT162b2 vaccine generated higher neutralizing titers against XBB.1.5, BA.2.86 and JN.1 compared to the bivalent BA.4/BA.5-adapted BNT162b2 vaccine.^40^ Here We also found that the 50% neutralizing GMTs in the sera of individuals one year after receiving the WSK-102C booster were 528, 483, 475 and 471 against JN.1, JN.1.13, KP.2 and KP.3 pseudoviruses, respectively. In contrast, the 50% neutralizing GMTs in the sera of individuals four months after receiving the WSK-102D booster were 1684, 1397, 1247 and 1298 against JN.1, JN.1.13, KP.2 and KP.3, respectively, all higher than those in the sera of individuals without boosters, indicating that current XBB.1.5 vaccines, such as WSK-V102C or WSK-V102D boosters, could still offer adequate protection against JN.1 and its sublineages.

As JN.1 subvariants, KP.2 and KP.3 have exhibited greater resistance to neutralizing antibodies compared to earlier Omicron subvariants, even including XBB.1.5 and JN.1, indicating that the newly introduced mutations play an important role in their immune evasion. Although studies have shown that the XBB.1.5 vaccine can still induce neutralizing antibodies capable of targeting both XBB.1.5 and JN.1, other research has found that antibody titers against JN.1, KP.2 and KP.3 are significantly lower compared to those against XBB.1.5.^41,42^ Recent data revealed that during the period when JN.1 was the dominant variant, the XBB.1.5 monovalent vaccine provided 54% protection (95% CI:46-60%) against symptomatic SARS-CoV-2 infection compared to unvaccinated individuals.^43^ Additionally, KP.2 showed a 1.45-fold greater ability to evade neutralizing antibodies in sera from individuals vaccinated with the COVID-19 bivalent vaccine booster, compared to the parental JN.1 variant.^44^ In line with these findings, our data showed that neutralization against JN.1.13, KP.2 and KP.3 was significantly decreased compared to that against JN.1 in sera from adults with WSK-V102C or WSK-V102D boosters. Furthermore, the GMTs of 50% neutralization against KP.2 and KP.3 were comparable, which is consistent with previous results that KP.2 and KP.3 subvariants exhibit similar immune evasion capabilities.^11^ Altogether, our findings highlight the enhanced neutralization resistance of JN.1 subvariants and underscore the importance of booster vaccines designed for currently circulating variants.

The emergence of new variants is a natural part of viral evolution, and JN.1 sublineages represent another chapter in the ongoing battle against COVID-19. Low levels of neutralizing antibodies in breakthrough infections against Omicron JN.1, KP.2 and KP.3 pose challenges in restricting the spread of JN.1 sublineages and maintaining population immunity. Previous studies have indicated seasonal patterns in the emergence and diversification of SARS-CoV-2 variants.^45^ Furthermore, research on the protective immunity triggered by seasonal coronaviruses, such as HCoV-NL63, HCoV-229E, HCoV-OC43 and HCoV-HKU1, all of which cause respiratory infections, has sought to identify common characteristics that might be applicable to other human coronaviruses.^46^ Their findings showed that immunity against seasonal coronaviruses is short-lasting, with reinfections occurring after the initial infection.^45^ Whether similar patterns will occur following infection with immune-evasive Omicron variants, such as KP.3.1.1 and XEC,^47^ remains unclear and requires further attention. Therefore, identification of the duration of acquired immunity and prediction of the future infection peaks driven by new variants is crucial. Nevertheless, vaccination remains the most potent tool in combating not just the currently circulating strain of SARS-CoV-2 but also its next variants. Previously approved vaccines, such as XBB.1.5 vaccines, still provide substantial protection. Moreover, these findings highlight ongoing research and development of vaccines specifically targeting new variants to keep pace with the evolution of SARS-CoV-2 variants.

## Materials and methods

### Materials

The Omicron sublineages pseudoviruses (BA.2.75, BF.7, BQ.1, XBB.1.5, XBB.1.9.1, XBB.1.16, XBB.2.3, EG.5.1, BA.2.86, JN.1, JN.1.13, KP.2, KP.3) expressing luciferase were purchased from Genomeditech (BA.2.75: GM-53320LV, BF.7: GM-55890LV, BQ.1: GM-57378LV, XBB.1.5: GM-59400LV, XBB.1.9.1: GM-75040LV, XBB.2.3: GM-75188LV, XBB.1.16: GM-76228LV, EG.5.1: GM-80273LV, BA.2.86: GM-80181LV, JN.1: GM-84672LV, JN.1.13: GM-87217LV, KP.2: GM-900007LV, KP.3: GM-87709LV). The One-Lumi™ II luciferase kits were purchased from Beyotime Biotechnology (RG056M).

### Serum samples

The blood samples were collected from adult participants in three separate periods, February 2023, October 2023 and February 2024, characterised by different vaccination and infection histories, in Chengdu, Sichuan Province, China. 120 samples were collected in February 2023, 2-3 months post a BA.5/BF.7 breakthrough infection in December 2022, but without any XBB.1.5-related booster. 120 samples were collected in October 2023. 99 serum samples were collected in February 2024, including 26 participants who had not received any booster for more than a year, 23 individuals who received a dose of WSK-V102C over a year, and 50 adults boosted with WSK-V102D over 4 months. Demographic Characteristics of the individuals were presented in detail in Table 1 and Table 2. All serum samples were isolated by centrifugation at 1500 rpm for 10 min at 4 °C. Sera were stored at −80 °C until use. These human participants involving research were reviewed and approved by the Ethics Committee Institution (No. 2022-1226). The written informed consent from all volunteers was obtained.

### Cell culture

ACE2 stably expressing 293 T cells (293 T/ACE2) were established and maintained in Dulbecco’s modified Eagle’s medium (DMEM, Gibco, USA), containing 10% fetal bovine serum (FBS, PAN-Biotech, Germany), 100 μg/ml streptomycin and 100 U penicillin (Gibco, USA), at 37 °C in a 5% CO_2_ environment.

### Pseudovirus neutralization assay

The pseudovirus neutralization assay was carried out according to our previous study.^48,49^ In brief, serum samples were prepared using a 1:3 serial dilution in 96-well plates with a final volume of 100 μl per well. The stock solutions of Omicron sublineages pseudoviruses (BA.2.75, BF.7, BQ.1, XBB.1.5, XBB.1.9.1, XBB.1.16, XBB.2.3, EG.5.1, BA.2.86, JN.1, JN.1.13, KP.2 and KP.3) were diluted by culture medium, and 50 μl of diluted pseudovirus solution was added into the above 96-well plates (Cat: WHB-96-03, WHB scientific) and incubated for 1 h at 37 °C. Then, 1.2 × 10^4^ human ACE2 receptor expressing HEK-293T (293 T/ACE2) cells with a volume of 100 μl were added to the wells containing the serum-virus mixture, followed by incubation at 37 °C for 48 h. Subsequently, the supernatant was removed and 100 μl of lysis reagent (Cat: RG056M, Beyotime Biotech) was added to the wells. The luminescence was measured by a multi-mode microplate reader (PerkinElmer, USA). The percentage of neutralization was calculated according to our previous method.^47^ The 50% neutralizing titers were calculated using GraphPad Prism 8.0 by a non-linear regression model.

### Phylogenetic analysis

A total of 59 amino acid sequences of the spike of SARS-CoV-2 variants and 45 nucleotide sequences of genome of JN.1 sublineages (Table S1) were downloaded from the NCBI database (www.ncbi.nlm.nih.gov/labs/virus) and GISAID database. The phylogenetic trees were constructed with the Molecular Evolutionary Genetics Analysis software (MEGA, version 11). Neighbor-Joining Tree statistical method was used for the phylogeny reconstruction analysis. Phylogeny test was through 1000 replications of Bootstrap test method and Maximum Composite Likelihood model. The trees were optimized using Interactive Tree of Life online tool (https://itol.embl.de).

### Statistical analysis

Statistical analyses were carried out using GraphPad Prism 8.0 (GraphPad software, MA, USA). Data are shown as geometric mean values ± SD. *P* values were calculated using unpaired or paired Student’s *t*-tests and two-way ANOVA for multiple comparisons, as detailed in each figure caption. *P* values < 0.05 were considered statistically significant.

## Supplementary information

Table S1

## Data Availability

All data and materials are available to the researchers once published.

## References

[1] Suryawanshi, R. K. et al. Limited cross-variant immunity from SARS-CoV-2 Omicron without vaccination. *Nature* **607**, 351–355 (2022). 35584773 10.1038/s41586-022-04865-0 PMC9279157

[2] Suzuki, R. et al. Attenuated fusogenicity and pathogenicity of SARS-CoV-2 Omicron variant. *Nature* **603**, 700–705 (2022). 35104835 10.1038/s41586-022-04462-1 PMC8942852

[3] He, X. et al. SARS-CoV-2 Omicron variant: Characteristics and prevention. *MedComm (2020)* **2**, 838–845 (2021). 34957469 10.1002/mco2.110 PMC8693031

[4] Kaku, Y. et al. Virological characteristics of the SARS-CoV-2 JN.1 variant. *Lancet Infect Dis* **24**, e82 (2024). 38184005 10.1016/S1473-3099(23)00813-7

[5] Yang, S. et al. Fast evolution of SARS-CoV-2 BA.2.86 to JN.1 under heavy immune pressure. *Lancet Infect Dis* **24**, e70–e72 (2024). 38109919 10.1016/S1473-3099(23)00744-2

[6] Kamble, P. et al. JN.1 variant in enduring COVID-19 pandemic: is it a variety of interest (VoI) or variety of concern (VoC)? *Horm Mol Biol Clin Investig* **45**, 49–53 (2024). 38622986 10.1515/hmbci-2023-0088

[7] Jony, M. H. K. et al. Emergence of SARS-CoV-2 Omicron sub-lineage JN.1 in Bangladesh. *Microbiol Resour Announc* **13**, e0013024 (2024). 38651907 10.1128/mra.00130-24 PMC11237766

[8] Branda, F. et al. Another variant another history: description of the SARS-CoV-2 KP.2 (JN.1.11.1.2) mutations. *Infect Dis (Lond)* **56**, 581–585 (2024). 38809158 10.1080/23744235.2024.2358383

[9] World Health Organization. *WHO tracking SARS-CoV-2 variants*, https://www.who.int/activities/tracking-SARS-CoV-2-variants. (2024).

[10] Kaku, Y. et al. Virological characteristics of the SARS-CoV-2 KP.2 variant. *Lancet Infect Dis* **24**, e416 (2024). 38782005 10.1016/S1473-3099(24)00298-6

[11] Kaku, Y. et al. Virological characteristics of the SARS-CoV-2 KP.3, LB.1, and KP.2.3 variants. *Lancet Infect Dis* **24**, e482–e483 (2024). 38945150 10.1016/S1473-3099(24)00415-8

[12] Kaku, Y. et al. Virological characteristics of the SARS-CoV-2 KP.3.1.1 variant. *Lancet Infect Dis* **24**, e609 (2024). 39159637 10.1016/S1473-3099(24)00505-X

[13] Global Initiative of Sharing All Influenza Data. *KP.2 Lineage Report*, https://outbreak.info/situation-reports?xmin=2023-12-06&xmax=2024-06-06&pango=KP.2. (2024).

[14] Global Initiative of Sharing All Influenza Data. *KP.3 Lineage Report*, https://outbreak.info/situation-reports?xmin=2024-01-22&xmax=2024-07-22&loc&pango=KP.3&selected. (2024).

[15] Centers for Disease Control and Prevention. *COVID Data Tracker*, https://covid.cdc.gov/covid-data-tracker/#variant-proportions. (2024).

[16] Global Initiative of Sharing All Influenza Data. *hCoV-19 variants dashboard* https://gisaid.org/phylodynamics/china-cn/. (2024).

[17] Harvey, W. T. et al. SARS-CoV-2 variants, spike mutations and immune escape. *Nat Rev Microbiol* **19**, 409–424 (2021). 34075212 10.1038/s41579-021-00573-0 PMC8167834

[18] Jangra, S. et al. SARS-CoV-2 spike E484K mutation reduces antibody neutralisation. *Lancet Microbe* **2**, e283–e284 (2021). 33846703 10.1016/S2666-5247(21)00068-9 PMC8026167

[19] Jian, F. et al. Convergent evolution of SARS-CoV-2 XBB lineages on receptor-binding domain 455-456 synergistically enhances antibody evasion and ACE2 binding. *PLoS Pathog* **19**, e1011868 (2023). 38117863 10.1371/journal.ppat.1011868 PMC10766189

[20] Ao, D., He, X., Hong, W. & Wei, X. The rapid rise of SARS-CoV-2 Omicron subvariants with immune evasion properties: XBB.1.5 and BQ.1.1 subvariants. *MedComm (2020)* **4**, e239 (2023). 36938325 10.1002/mco2.239 PMC10015854

[21] Wang, Q. et al. Antigenicity and receptor affinity of SARS-CoV-2 BA.2.86 spike. *Nature* **624**, 639–644 (2023). 37871613 10.1038/s41586-023-06750-w

[22] Wang, X., Lu, L. & Jiang, S. SARS-CoV-2 evolution from the BA.2.86 to JN.1 variants: unexpected consequences. *Trends Immunol* **45**, 81–84 (2024). 38302341 10.1016/j.it.2024.01.003

[23] Qu, P. et al. Enhanced neutralization resistance of SARS-CoV-2 Omicron subvariants BQ.1, BQ.1.1, BA.4.6, BF.7, and BA.2.75.2. *Cell Host Microbe* **31**, 9–17.e13 (2023). 36476380 10.1016/j.chom.2022.11.012 PMC9678813

[24] Focosi, D., Spezia, P. G., Gueli, F. & Maggi, F. The Era of the FLips: How Spike Mutations L455F and F456L (and A475V) Are Shaping SARS-CoV-2 Evolution. *Viruses* **16**, 3 (2023). 38275938 10.3390/v16010003 PMC10818967

[25] Chand, G. B., Banerjee, A. & Azad, G. K. Identification of twenty-five mutations in surface glycoprotein (Spike) of SARS-CoV-2 among Indian isolates and their impact on protein dynamics. *Gene Rep* **21**, 100891 (2020). 33015411 10.1016/j.genrep.2020.100891 PMC7521409

[26] Chakraborty, C. & Bhattacharya, M. FLip mutations (L455F + F456L) in newly emerging VOI, JN.1: Its antibody and immune escape. *Int Immunopharmacol* **133**, 112146 (2024). 38677090 10.1016/j.intimp.2024.112146

[27] Ba, Z. et al. Reflections on the dynamic zero-COVID policy in China. *Prev Med Rep* **36**, 102466 (2023). 38116286 10.1016/j.pmedr.2023.102466 PMC10728318

[28] Ioannidis, J. P. A., Zonta, F. & Levitt, M. What Really Happened During the Massive SARS-CoV-2 Omicron Wave in China? *JAMA Intern Med* **183**, 633–634 (2023). 37184847 10.1001/jamainternmed.2023.1547 PMC10404134

[29] Wang, X. et al. Robust neutralization of SARS-CoV-2 variants including JN.1 and BA.2.87.1 by trivalent XBB vaccine-induced antibodies. *Signal Transduct Target Ther* **9**, 123 (2024). 38724561 10.1038/s41392-024-01849-6 PMC11082144

[30] Ito, J. et al. Convergent evolution of SARS-CoV-2 Omicron subvariants leading to the emergence of BQ.1.1 variant. *Nat Commun* **14**, 2671 (2023). 37169744 10.1038/s41467-023-38188-z PMC10175283

[31] Faraone, J. N. et al. Immune evasion and membrane fusion of SARS-CoV-2 XBB subvariants EG.5.1 and XBB.2.3. *Emerg Microbes Infect* **12**, 2270069 (2023). 37819267 10.1080/22221751.2023.2270069 PMC10606793

[32] Raisinghani, N. et al. AlphaFold2 Modeling and Molecular Dynamics Simulations of the Conformational Ensembles for the SARS-CoV-2 Spike Omicron JN.1, KP.2 and KP.3 Variants: Mutational Profiling of Binding Energetics Reveals Epistatic Drivers of the ACE2 Affinity and Escape Hotspots of Antibody Resistance. *Viruses* **16**, 1458 (2024). 39339934 10.3390/v16091458 PMC11437503

[33] Groenheit, R. et al. Rapid emergence of omicron sublineages expressing spike protein R346T. *Lancet Reg Health Eur* **24**, 100564 (2023). 36533118 10.1016/j.lanepe.2022.100564 PMC9735319

[34] Li, P. et al. Distinct patterns of SARS-CoV-2 BA.2.87.1 and JN.1 variants in immune evasion, antigenicity, and cell-cell fusion. *mBio* **15**, e0075124 (2024). 38591890 10.1128/mbio.00751-24 PMC11077997

[35] Qu, P. et al. Immune evasion, infectivity, and fusogenicity of SARS-CoV-2 BA.2.86 and FLip variants. *Cell* **187**, 585–595 e586 (2024). 38194968 10.1016/j.cell.2023.12.026 PMC10872432

[36] Huiberts, A. J. et al. Effectiveness of Omicron XBB.1.5 vaccine against infection with SARS-CoV-2 Omicron XBB and JN.1 variants, prospective cohort study, the Netherlands, October 2023 to January 2024. *Euro Surveill*. **29**, (2024). 10.2807/1560-7917.ES.2024.29.10.2400109 PMC10986669 38456217

[37] Wang, Q. et al. XBB.1.5 monovalent mRNA vaccine booster elicits robust neutralizing antibodies against XBB subvariants and JN.1. *Cell Host Microbe* **32**, 315–321.e313 (2024). 38377995 10.1016/j.chom.2024.01.014 PMC10948033

[38] Willett, B. J. et al. Omicron BA.2.86 cross-neutralising activity in community sera from the UK. *Lancet* **402**, 2075–2076 (2023). 37952549 10.1016/S0140-6736(23)02397-8

[39] Khan, K. et al. Evolution and neutralization escape of the SARS-CoV-2 BA.2.86 subvariant. *Nat Commun* **14**, 8078 (2023). 38057313 10.1038/s41467-023-43703-3 PMC10700484

[40] Gayed, J. et al. Immunogenicity of the Monovalent Omicron XBB.1.5-Adapted BNT162b2 COVID-19 Vaccine against XBB.1.5, BA.2.86, and JN.1 Sublineages: A Phase 2/3 Trial. *Vaccines (Basel)* **12**, 734 (2024). 39066372 10.3390/vaccines12070734 PMC11281410

[41] Chalkias, S. et al. Safety and Immunogenicity of XBB.1.5-Containing mRNA Vaccines. *medRxiv*, 2023.2008.2022.23293434, (2023).

[42] Gillot, C. et al. Neutralizing antibodies against KP.2 and KP.3: why the current vaccine needs an update. *Clin Chem Lab Med* **10**, 1515 (2024). 10.1515/cclm-2024-0919 39147380

[43] Link-Gelles, R. et al. Early Estimates of Updated 2023-2024 (Monovalent XBB.1.5) COVID-19 Vaccine Effectiveness Against Symptomatic SARS-CoV-2 Infection Attributable to Co-Circulating Omicron Variants Among Immunocompetent Adults - Increasing Community Access to Testing Program, United States, September 2023-January 2024. *MMWR Morb Mortal Wkly Rep* **73**, 77–83 (2024). 38300853 10.15585/mmwr.mm7304a2 PMC10843065

[44] Li, P. et al. Neutralization escape, infectivity, and membrane fusion of JN.1-derived SARS-CoV-2 SLip, FLiRT, and KP.2 variants. *Cell Rep* **43**, 114520 (2024). 39024099 10.1016/j.celrep.2024.114520 PMC11430188

[45] Becerra-Artiles, A. et al. Immunopeptidome profiling of human coronavirus OC43-infected cells identifies CD4 T-cell epitopes specific to seasonal coronaviruses or cross-reactive with SARS-CoV-2. *PLoS Pathog* **19**, e1011032 (2023). 37498934 10.1371/journal.ppat.1011032 PMC10409285

[46] Edridge, A. W. D. et al. Seasonal coronavirus protective immunity is short-lasting. *Nat Med* **26**, 1691–1693 (2020). 32929268 10.1038/s41591-020-1083-1

[47] Arora, P. et al. Impact of JN.1 booster vaccination on neutralisation of SARS-CoV-2 variants KP.3.1.1 and XEC. *Lancet Infect Dis* **24**, e732–e733 (2024). 39522531 10.1016/S1473-3099(24)00688-1

[48] Yang, J. et al. A vaccine targeting the RBD of the S protein of SARS-CoV-2 induces protective immunity. *Nature* **586**, 572–577 (2020). 32726802 10.1038/s41586-020-2599-8

[49] He, C. et al. Spike protein of SARS-CoV-2 Omicron (B.1.1.529) variant have a reduced ability to induce the immune response. *Signal Transduct Target Ther* **7**, 119 (2022). 35397623 10.1038/s41392-022-00980-6 PMC8994023

