# Supplementary material for: Recombinant XBB.1.5 boosters induce robust neutralization against KP.2- and KP.3-included JN.1 sublineages
Source: Signal Transduct Target Ther. 2025 Jan 27;10:47. doi: 10.1038/s41392-025-02139-5 (PMC11772742; doi:10.1038/s41392-025-02139-5)
Supplement: Supplementary file 1 — Table S1 [file 41392_2025_2139_MOESM1_ESM.docx]

Supplementary Materials for

Recombinant XBB.1.5 booster induces robust neutralization against KP.2- and KP.3-included JN.1 sublineages

Jingyun Yang^1^†, Xuemei He^1^†, Huashan Shi^1^†, Cai He^1^†, Hong Lei^1^†, Heng He^1^†, Li Yang^1^, Wei Wang^1^, Guobo Shen^1^, Jinliang Yang^1^, Zhiwei Zhao^1^, Xiangrong Song^1^, Zhenling Wang*, Guangwen Lu*, Jiong Li*, Yuquan Wei^1^*

Correspondence to: wangzhenling@scu.edu.cn, lugw@scu.edu.cn, lijionghh@scu.edu.cn, and yqwei@vip.sina.com

**This PDF file includes:**

Table S1

Table S1.

Information of amino acid sequences and nucleotide sequences downloaded from the NCBI and GISAID databases.

| No. | NCBI/GISAID ID | Country | Collection date | Pangolin |
| --- | --- | --- | --- | --- |
| Amino acid sequences | | | | |
| 1 | XLJ72221.1 | USA | 2022/4/21 | BA.2 |
| 2 | XLU40546.1 | France | 2022/1/31 | BA.2 |
| 3 | XLU40650.1 | France | 2022/2/7 | BA.2 |
| 4 | XLV52475.1 | USA | 2022/3/21 | BA.2 |
| 5 | XLW54941.1 | USA | 2022/4/4 | BA.2 |
| 6 | XLW65778.1 | USA | 2022/7/11 | BA.2 |
| 7 | BFN80727.1 | Japan | 2022/7/27 | BA.2.75 |
| 8 | WFI72407.1 | USA | 2022/7/23 | BA.2.75 |
| 9 | WGH48125.1 | India | 2022/7/30 | BA.2.75 |
| 10 | WGH48220.1 | India | 2022/8/9 | BA.2.75 |
| 11 | XKT61232.1 | USA | 2022/11/10 | BA.2.75 |
| 12 | WWQ06071.1 | USA | 2023/12/22 | BA.2.86 |
| 13 | WYJ49252.1 | USA | 2024/3/11 | BA.2.86 |
| 14 | XAT77441.1 | USA | 2023/10/18 | BA.2.86 |
| 15 | XDE98613.1 | USA | 2023/10/26 | BA.2.86 |
| 16 | XDF74460.1 | USA | 2024/2/19 | BA.2.86 |
| 17 | UPU63007.1 | USA | 2022/4/16 | BA.4 |
| 18 | UQV39663.1 | USA | 2022/5/3 | BA.4 |
| 19 | URC97430.1 | USA | 2022/5/12 | BA.4 |
| 20 | URD24205.1 | USA | 2022/5/6 | BA.4 |
| 21 | UPU09668.1 | USA | 2022/4/15 | BA.5 |
| 22 | URG11549.1 | USA | 2022/5/12 | BA.5 |
| 23 | URH69818.1 | Bahrain | 2022/5/14 | BA.5 |
| 24 | URN53666.1 | South Africa | 2022/4/28 | BA.5 |
| 25 | URO10800.1 | USA | 2022/5/18 | BA.5 |
| 26 | XKI00319.1 | USA | 2024/2/8 | JN.1 |
| 27 | XKS37984.1 | USA | 2024/1/24 | JN.1 |
| 28 | XKS38569.1 | USA | 2024/1/4 | JN.1 |
| 29 | UUH56739.1 | USA | 2022/7/19 | BF.7 |
| 30 | UUR59598.1 | USA | 2022/7/27 | BF.7 |
| 31 | UVB68683.1 | USA | 2022/7/25 | BF.7 |
| 32 | UVD64009.1 | USA | 2022/8/2 | BF.7 |
| 33 | WAH86792.1 | USA | 2022/11/25 | XBB.1.5 |
| 34 | WAI91846.1 | USA | 2022/11/26 | XBB.1.5 |
| 35 | WAN20920.1 | USA | 2022/11/26 | XBB.1.5 |
| 36 | WAN21726.1 | USA | 2022/11/27 | XBB.1.5 |
| 37 | WAN24888.1 | USA | 2022/11/27 | XBB.1.5 |
| 38 | WBY68420.1 | USA | 2023/1/9 | XBB.2.3 |
| 39 | WCS57654.1 | USA | 2023/1/23 | XBB.2.3 |
| 40 | WEW91142.1 | USA | 2023/3/12 | XBB.2.3 |
| 41 | WFD68080.1 | USA | 2023/3/22 | XBB.2.3 |
| 42 | WCM02109.1 | USA | 2023/1/23 | XBB.1.16 |
| 43 | WEI68834.1 | USA | 2023/3/4 | XBB.1.16 |
| 44 | WGH32986.1 | USA | 2023/4/12 | XBB.1.16 |
| 45 | WGH58293.1 | USA | 2023/4/9 | XBB.1.16 |
| 46 | WGP26425.1 | USA | 2023/4/13 | EG.5.1 |
| 47 | WHT82823.1 | USA | 2023/5/8 | EG.5.1 |
| 48 | WIN99655.1 | USA | 2023/5/30 | EG.5.1 |
| 49 | WIY84495.1 | USA | 2023/6/2 | EG.5.1 |
| 50 | WKK53590.1 | USA | 2023/7/5 | HV.1 |
| 51 | WKY40789.1 | USA | 2023/7/20 | HV.1 |
| 52 | WLM01235.1 | USA | 2023/7/8 | HV.1 |
| 53 | WLS41433.1 | USA | 2023/7/30 | HV.1 |
| 54 | WLS41911.1 | USA | 2023/7/31 | HV.1 |
| 55 | WMS63871.1 | USA | 2023/8/15 | HK.3 |
| 56 | WMV02984.1 | USA | 2023/8/23 | HK.3 |
| 57 | WNC81540.1 | USA | 2023/8/30 | HK.3 |
| 58 | WND62797.1 | USA | 2023/8/22 | HK.3 |
| 59 | WNF02286.1 | USA | 2023/9/7 | HK.3 |
| Nucleotide sequences | | | | |
| 60 | PP485501.1 | USA | 2024/1/5 | JN.1.13 |
| 61 | PP455100.1 | USA | 2024/2/10 | JN.1.13 |
| 62 | PP406095.1 | USA | 2024/1/10 | JN.1.13 |
| 63 | OY785795.1 | Denmark | 2023/11/6 | JN.1.13 |
| 64 | OY995719.1 | Denmark | 2023/12/11 | JN.1.13 |
| 65 | OZ019555.1 | UK | 2024/2/9 | JN.1.5 |
| 66 | PP406275.1 | UK | 2024/1/16 | JN.1.5 |
| 67 | OZ011983.1 | UK | 2024/1/26 | JN.1.5 |
| 68 | OZ013602.1 | Denmark | 2023/12/25 | JN.1.5 |
| 69 | OZ010262.1 | UK | 2024/1/17 | JN.1.5 |
| 70 | OY994240.1 | Denmark | 2023/12/11 | JN.7 |
| 71 | OZ014221.1 | Denmark | 2024/1/8 | JN.7 |
| 72 | OZ014199.1 | Denmark | 2023/12/25 | JN.7 |
| 73 | OZ014039.1 | Denmark | 2023/12/25 | JN.7 |
| 74 | OZ013772.1 | Denmark | 2024/1/8 | JN.7 |
| 75 | PP799339.1 | USA | 2024/1/3 | JN.1.7 |
| 76 | PP799299.1 | USA | 2023/12/28 | JN.1.7 |
| 77 | PP796990.1 | USA | 2023/12/29 | JN.1.7 |
| 78 | PP794095.1 | USA | 2023/12/23 | JN.1.7 |
| 79 | PP763864.1 | USA | 2024/3/15 | JN.1.7 |
| 80 | PP346560.1 | USA | 2024/2/19 | JN.1.18 |
| 81 | PP455076.1 | USA | 2024/2/15 | JN.1.18 |
| 82 | PP466652.1 | USA | 2024/3/11 | JN.1.18 |
| 83 | PP410325.1 | USA | 2024/2/28 | JN.1.18 |
| 84 | PP432704.1 | USA | 2024/3/4 | JN.1.18 |
| 85 | EPI_ISL_19110137 | Canada | 2024/4/22 | LB.1 |
| 86 | EPI_ISL_19118241 | Canada | 2024/4/28 | LB.1 |
| 87 | EPI_ISL_19095978 | Singapore | 2024/4/24 | LB.1 |
| 88 | EPI_ISL_19095882 | Singapore | 2024/4/19 | LB.1 |
| 89 | EPI_ISL_19066736 | Canada | 2024/4/12 | LB.1 |
| 90 | EPI_ISL_19118962 | Canada | 2024/4/17 | KP.3 |
| 91 | EPI_ISL_19119092 | Canada | 2024/4/25 | KP.3 |
| 92 | EPI_ISL_19119144 | Canada | 2024/4/25 | KP.3 |
| 93 | EPI_ISL_19118227 | Canada | 2024/4/28 | KP.3 |
| 94 | EPI_ISL_19118150 | Canada | 2024/4/22 | KP.3 |
| 95 | EPI_ISL_19118230 | Canada | 2024/4/29 | KP.2 |
| 96 | EPI_ISL_19122563 | Australia | 2024/4/20 | KP.2 |
| 97 | EPI_ISL_19110163 | Canada | 2024/4/25 | KP.2 |
| 98 | EPI_ISL_19109566 | Canada | 2024/4/15 | KP.2 |
| 99 | EPI_ISL_19110267 | Japan | 2024/4/9 | KP.2 |
| 100 | EPI_ISL_19044112 | Canada | 2024/3/18 | KP.1.1 |
| 101 | EPI_ISL_19033226 | Singapore | 2024/3/20 | KP.1.1 |
| 102 | EPI_ISL_19035144 | France | 2024/3/14 | KP.1.1 |
| 103 | EPI_ISL_19027267 | Canada | 2024/3/9 | KP.1.1 |
| 104 | EPI_ISL_18987166 | Singapore | 2024/3/11 | KP.1.1 |
